# Dimensional Structure and Preliminary Results of the External Constructs of the Schizophrenia Coping Oral Health Profile and Index (SCOOHPI)

**DOI:** 10.3390/ijerph182312413

**Published:** 2021-11-25

**Authors:** Francesca Siu-Paredes, Nathalie Rude, Ines Rouached, Corinne Rat, Rachid Mahalli, Wissam El-Hage, Katherine Rozas, Frédéric Denis

**Affiliations:** 1 Faculty of Dentistry, Champagne-Ardenne of Reims University Hospital, 51100 Reims, France; francesca.siu-paredes@univ-reims.fr (F.S.-P.); rozaskatherine@hotmail.com (K.R.); 2 UR 481 Laboratoire de Recherches Intégratives en Neurosciences et Psychologie Cognitive, University of Bourgogne Franche-Comté, 25000 Besançon, France; nathalie.retel-rude@univ-fcomte.fr (N.R.); ines.rouached@edu.univ-fcomte.fr (I.R.); 3 Clinical Research Unit, La Chartreuse Hospital Center, 21000 Dijon, France; Corinne.Rat@chlcdijon.fr; 4 Department of Odontology, Tours University Hospital Center, 37000 Tours, France; rachid.mahalli@etu.univ-tours.fr; 5 U1253, iBrain, CIC1415, Inserm, University Hospital Centre, Université de Tours, 37000 Tours, France; wissam.elhage@univ-tours.fr; 6 Faculty of Dentistry, Nantes University, 44000 Nantes, France; 7 EA 75-05 Education, Ethics, Health, Faculty of Medicine, François-Rabelais University, 37000 Tours, France

**Keywords:** schizophrenia, psychometric, coping strategies, oral health

## Abstract

The Schizophrenia Coping Oral Health Profile and Index (SCOOHPI) was developed to assess oral health coping strategies in people with schizophrenia. We show that the difficulty and discrimination indices of 18 items, selected for the final version, are acceptable according to the Rasch model, as are the inter-item (0.25) and inter-score (α = 0.85) correlations. This scale can be considered as an index, giving a global score between 0 and 72, with a Likert scale with five response modalities. This is also a profile with the following three dimensions of coping-related oral health, emerging independently of each other: (1) physical well-being strategies (α = 0.72); (2) moral well-being strategies (α = 0.60); (3) access strategies for oral well-being (α = 0.79). The sub-scores, ranging from 0 to 24, specify populations focused on the themes of coping strategies that may be most affected, depending on the subject’s characteristics and their clinical oral health status. The validation study of this scale is still in progress, to evaluate the reproducibility of the results, sensitivity to change, and reliability for other populations of people with schizophrenia.

## 1. Introduction

People with systemic disorders (such as diabetes, cardiovascular disease, or kidney disease) or connective tissue diseases (such as rheumatoid arthritis or lupus erythematosus) often suffer from a deteriorated psychological state. Anxiety, stress or depressive disorders resulting from poor physical health also impact oral health, which, in this context, is often neglected, thus it rapidly deteriorates due to a lack of self-care or regular dental visits [1].

Similarly, people with severe mental illnesses, such as schizophrenia, often exhibit both health and lifestyle behaviors that lead to poor oral health and dental disease [2]. In addition to the oral health side effects of antipsychotics, people with schizophrenia (PWS) neglect their own care [3,4]. This seems to be mainly influenced by the negative symptoms of schizophrenia, such as the lack of initiative, lack of interest in personal health, social withdrawal, and lack of motivation [5,6].

Conversely, poor oral health impacts self-esteem and the ability of PWS to engage in recovery [6].

From a public health perspective, it is important to develop effective oral health promotion or support programs for these people, and to evaluate them using reliable and valid tools. One difficulty in measuring perceived health in those with mental disorders is dissociation from the reality of the illness, intensity of the response, and adequacy of their perception [7]. PWS tend to have generalized cognitive impairment, affecting communication and social skills [8]. Cognitive impairment may be associated with stereotypical behavior, a symptom characterized by repetitive and functionless motor behavior [9]. These factors put PWS at risk of social dysfunction or loss of self-esteem, leading to isolation and victimization [10].

In this context, new strategies working towards improved oral health and managing the underlying psychological mechanisms, promoting adherence to these strategies, remain underexplored.

Folkman and Moskowitz [11] define coping as the thoughts and behaviors used to deal with stressful situations. Coping must be understood as an explanatory concept for the variability in the response to a stressor. Coping strategies to stressors are, therefore, specific to each situation and to each person. In other words, beyond measuring perceived health, it is important to know how these people concretely organize themselves in terms of, for example, handling dental pain or organizing themselves to be regularly followed up by a dentist. It is also important to know the emotional impact an oral disorder can have. The challenge for carers is to better understand the coping strategies used according to the experiences, perceptions, and representations of each PWS.

As such, a specific tool was developed to evaluate oral health coping strategies in PWS. This scale was named the Schizophrenia Coping Oral Health Profile (SCOOHP) [12]. The provisional, self-administered scale consists of 23 items and two dimensions, with positive coping of 15 items and negative coping of eight items. The SCOOHP aims to identify coping strategies used by PWS for oral health problems and blockages,—while caring for one’s health (negative coping) or facilitating “good” behaviors to optimize oral health (positive coping)—for target care and protocols that improve their oral health. Although this draft scale has good acceptability and consistent reliability (α = 0.59), the dimensional and external structure of this scale must be validated from a psychometric point of view.

Subjective measurement scales must provide accurate, valid, interpretable and scientifically robust data for population health assessment before they are considered suitable. The performance of the results of these measures is mainly due to the reliability and validity of these scales [13]. Reliability is the ability to reproduce a consistent result over time and space, or across different observers, and is one of the main quality criteria of an instrument [14,15]; it mainly refers to the validation of the dimensional structure of the scale [14,15]. Validity, or the study of external constructs, refers to whether a tool measures exactly what it is supposed to measure [16]; for example, criterion validity is the relationship between the scale score to be validated and an external criterion [17]. This criterion should be a widely accepted measure, with the same characteristics as the assessment tool, and should be considered the ‘gold standard’ where it exists [17]. This study aims to validate the dimensional structure of the provisional SCOOHP scale, and to explore its external structure.

## 2. Materials and Methods

### 2.1. Research Design

The SCOOHP questionnaire was developed between June 2016 and November 2018 in a monocentric qualitative study carried out at the Hospital Centre la Chartreuse in Dijon (France). This study involved 34 people (24 PWS and 10 health professionals) [12]. The PWS questionnaire was developed and structured with data collected during semi-structured interviews with 20 PWS and six health professionals, and two focus groups with eight different people (1 FG with 4 people with PWS and 1 FG with health professionals). Only items related to the concept of oral health adaptation were retained for the construction of this questionnaire [12]. A psychometric validation study of this scale has been underway since 2018, with a large sample of PWS recruited from five French hospitals (Dijon, Tours, Reims, Millau and Paris). The study was registered with www.ClinicalTrials.gov (accessed on 1 October 2021) under the number NCT03699501.

Patients were referred to the study investigator through the health professional designated as responsible for the patient’s mental health care. They were informed by the investigator of the nature of the research, its objectives, methodology, duration, expected benefits, constraints and foreseeable risks, in accordance with Article L1122-1 of the Public Health Code. PWS were informed that their data would be computerized, confidential and processed anonymously, and that they could access and rectify it at any time.

**Figure 1:**
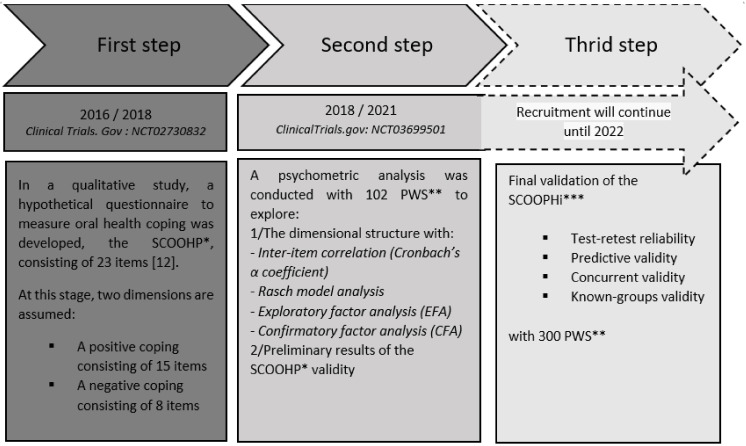
Flow chart of the study. SCOOHP*: Schizophrenia Coping Oral Health profile; PWS**: Persons with schizophrenia; SCOOHPI***: Schizophrenia Coping Oral Health profile and Index [12].

Below, Figure 1 summarizes the research design.

### 2.2. Analysis of the Dimensional Structure

To explore the dimensional structure of the provisional SCOOHP questionnaire and validate its final version, we used (1) inter-item correlation analysis and Cronbach’s α coefficient, (2) Rasch model analysis, (3) exploratory factor analysis (EFA), and (4) confirmatory factor analysis (CFA).

#### 2.2.1. Inter-Item Correlation and Cronbach’s α Coefficient

Internal consistency was assessed with an inter-item correlation of global scores and Cronbach’s α coefficient values. Such correlations examine the extent to which scores on one item on a scale are related to scores on all other items on that scale. Ideally, the average inter-item correlation for a set of items is between 0.20 and 0.40. When the values are less than 0.20, the items may not represent the same content domain. Cronbach’s α values greater than 0.75 indicate excellent reliability, while values between 0.40 and 0.75 indicate fair to good reliability, and values less than 0.40 indicate poor reliability [18,19]

#### 2.2.2. Rasch Model Analysis

The Rasch model was used to examine the extent to which the interim SCOOHP scale functioned as a measure of oral health coping strategies in individuals with schizophrenia. Given that the goal was to refine the draft instrument, the analysis was primarily used to highlight items or scoring categories that had a substantial “mismatch” with the model, suggesting that they might not usefully contribute to, or may even degrade, the instrument’s performance as a measurement system, which could be removed [20].

#### 2.2.3. Exploratory Factor Analysis (EFA)

EFA is often used in addition to Rasch analysis to explore characteristics of an instrument, guide its development, and verify that the items measure the same trait [21]. If this is not the case (Kaiser–Meyer–Olkin, KMO, value > 0.6 and Bartlett test results show significant sphericity), it may be necessary to divide the scale into subscales or delete items [21,22].

#### 2.2.4. Analysis of the Confirmatory Factor Analysis (CFA)

A CFA was performed to test stability of factor structure of the final version of the SCOOHP scale, accomplished by investigating the goodness of fit using chi-square/DF; root mean square error of approximation (RMSEA), comparative fit index (CFI), and IFI (incremental fit index) [23].

### 2.3. Preliminary Result of the SCOOHP Validity

To study the validity of the SCOOHP scale, it is first assumed that there are correlations between scores of the Brief-COPE scale and the SCOOHP scale. The Brief-COPE scale, the French version of which was validated by Muller and Siptz [24], is designed to assess the usual way of coping with stressors in daily life. The Brief-COPE scale is also used to assess the particular ways in which individuals cope with a specific stressful situation.

### 2.4. Ethical Considerations

This study, named “Quality bis”, was approved by the Committee for the Protection of Persons of the Ile de France region (registration number: 2018-A02043-52). After participants had a complete description of the study, informed consent was obtained from each participant, or from the legal guardians of individuals under guardianship. In the latter case, the patient’s legal guardian(s) signed the informed consent.

### 2.5. Data Analysis

The analysis was conducted using software package R^®^ (Bell Laboratories, New Providence, NJ, USA).

## 3. Results

### 3.1. Participants’ General Characteristics

The PWS who agreed to participate were over 18 years old and had received a diagnosis of schizophrenia (according to the International Classification of Diseases, 10th Revision: ICD-10) [25]. The recruited PWS were psychically stable according to a psychiatric evaluation. PWS who could not understand or had a poor understanding of French were excluded.

For the 102 PWS who participated in the “Quality bis” study, there were less than 10% missing answers to the questionnaires. The participants’ characteristics were described in a previous study [12]. The average age of the participants was 40.7 ± 11.5 years, 67.7% were male, and most were single (74.5%). One in two were smokers (45.1%).

### 3.2. The Psychometric Properties of the Provisional SCOOHP Questionnaire (23 Items)

#### 3.2.1. Item Correlation with the Global Score

The correlation of each item (1 to 23) of SCOOHP with a global score is presented in Table 1.

#### 3.2.2. Rasch Model Analysis

The mean of the response modality values was between two and four, with standard deviations between one and two for each item. The modalities were coded from zero to four, (Table 2)

#### 3.2.3. Exploratory Factor Analysis (EFA)

**Figure 2:**
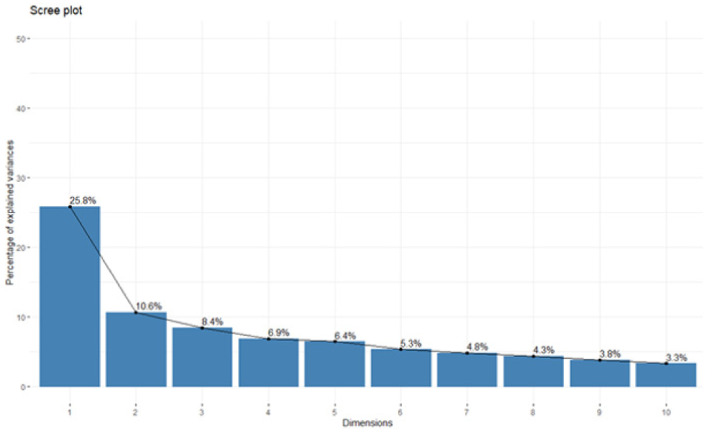
SCOOHP (23 items) eigenvalues graph.

EFA was performed on 23 items to search for possible dimensions and confirm the exclusion of the five items previously removed after the Rasch analysis. Figure 2 allows us to determine which factor items (or factorial dimensions) are best represented.

**Figure 3:**
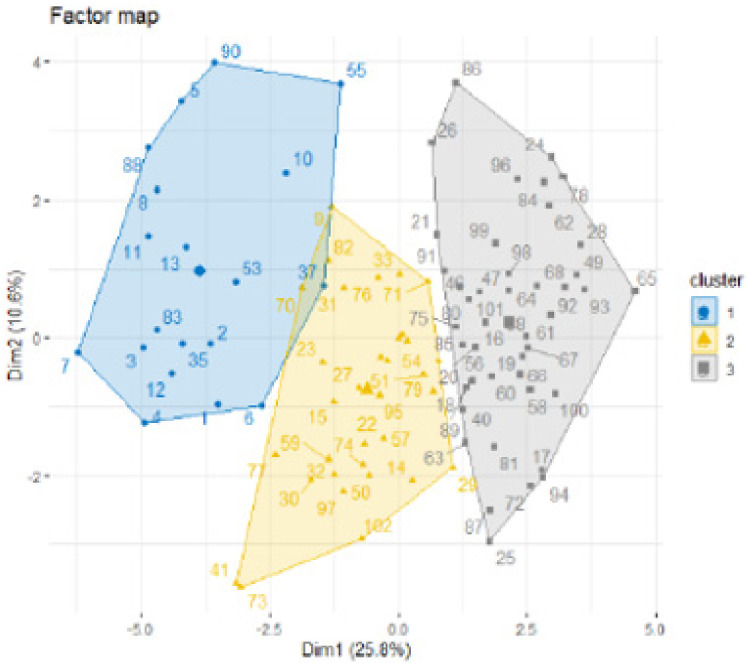
The individual factor map for the SCOOHP scale with 23 items.

The first axis of the principal component analysis (PCA) concentrates 25.8% of the information. The second, third, fourth, fifth, and sixth axes contained 10.60%, 8.4%, 6.9%, 6.4%, and 5.3%, respectively. We use approximately 63.4% of total variance, in the first six axes, retained for the confirmatory factor analysis. In Figure 3, we show the individuals’ factor maps for the SCOOHP scale with 23 items.

#### 3.2.4. Analysis of the Confirmatory Factor Analysis (CFA)

**Figure 4:**
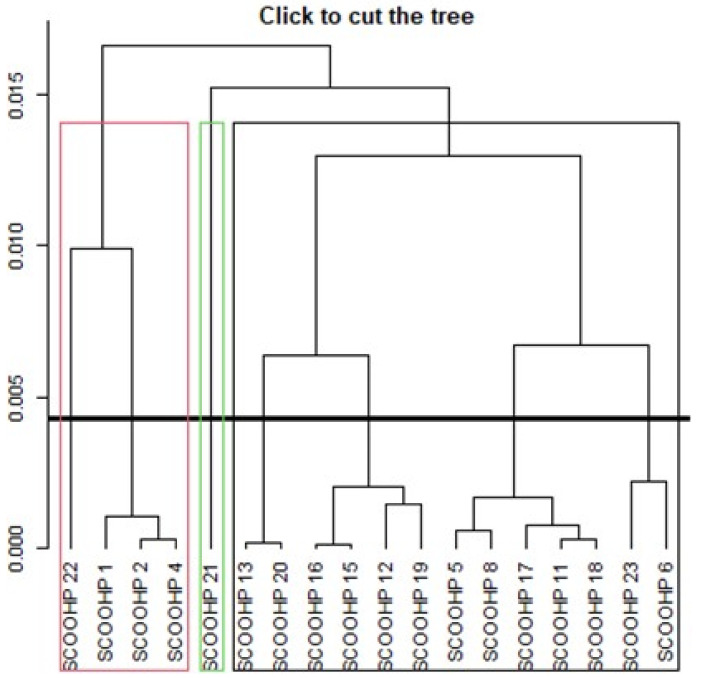
Individual items dendrogram for the SCOOHP scale with 18 items.

Since the factorial analysis did not allow us to obtain sub-themes (dimensions), we hypothesized about the grouping of items according to the proximity of conceptual contents. Figure 4 shows that three groups were formed with 18 items.

The SCOOHP items 1, 2, 4, and 22 are grouped into group 1, while items 5, 6, 8, 11, 12, 13, 15, 16, 17, 18, 19, 20, and 23 are in group 2. SCOOHP item 21 is in group 3. The three clusters did not match our conceptual grouping, but the Cronbach’s α of the three clusters, obtained by the hierarchical method, are satisfactory. Cronbach’s α was 0.669 for group 1 and 0.872 for group 2. Item 21 was isolated. Since an isolated item does not allow us to calculate a Cronbach’s α, we searched for different dimensions by a conceptual approach, by looking for the grouping of items according to the same sub-concept, i.e., according to the proximity of the items for coping strategies. In light of the analysis, and in order to obtain more precision in the measurement, we looked for dimensions that would allow us to have more precise sub-scores. Three dimensions emerged following logical or hypothetical grouping of the items according to their conceptual proximity by theme. We reorganized the SCOOHP items into the following three dimensions: physical well-being strategies (items 5, 8, 11, 17, 19, and 21); moral well-being strategies (items 1, 2, 4, 20, 22, and 23); oral well-being strategies (items 6, 12, 13, 15, 16, and 18). 

In light of these results, we have named the 18-item SCOOHP the Schizophrenia Coping Oral Health Profile and Index (SCOOHPI).

### 3.3. The Psychometric Properties of the SCOOHPI Questionnaire (18 Items)

#### 3.3.1. Inter-Item Correlation

The correlation of each item (1 to 18) of SCOOHPI with a global score is presented in Table 3. In this table, the numbering of 18 items is the same as that of SCOOHP.

#### 3.3.2. Rasch Model Analysis of SCOOHPI

Upon the removal of items 3, 7, 9, 10, and 14, the average inter-item correlation increased from 0.15 to 0.25, and the Cronbach’s coefficient increased from 0.80 to 0.85. In Table 4, Table 5 and Table 6, we expose the results of the Rasch analysis of the three presupposed dimensions linked to the conceptual proximity between items.

#### 3.3.3. Preliminary Result of the SCOOHPI Validity

Table 7 shows the correlations between each dimension of the SCOOHPI (18 items) and the items of the Brief-COPE scale.

## 4. Discussion

The SCOOHPI scale was developed to assess oral health coping strategies in PWS. We show that the discrimination indices of each of the 18 items are acceptable, as per the Rasch model, as are the inter-item (0.25) and inter-score correlations (α = 0.85). This scale can be considered an index, with a global score between 0 and 72, and a Likert scale with five response modalities from 0 to 4. It is also a profile of the following three dimensions of coping related to oral health, which emerged independently of each other: physical well-being strategies (α = 0.72); moral well-being strategies (α = 0.60); access strategies towards oral well-being (α = 0.79). The three sub-scores, ranging from 0 to 24, make it possible to specify the populations observed to the coping strategies that may be most affected, depending on the characteristics of the subjects and their clinical health status. With six items per dimension, it is possible to obtain comparable scores of patients’ coping “levels” according to each of the three “physical”, “moral” and “oral” dimensions, and also according to sex, age, disease grade, and type of treatment.

We also observed a discrepancy between presupposed coping strategies (positive or negative) and the final number of dimensions of the SCOOHPI scale after internal structure validation. Three different dimensions allow for a dynamic assessment of coping processes, by assessing the ability to engage in different types of coping responses over time, in response to environmental. This approach is consistent with the “perceived capacity” model of Kato et al. [26]. This work allowed us to challenge our original assumptions from a biomedical model regarding the consequences of oral health problems, rather than the person affected by those problems [27]. These results confirm a need to go beyond linear conceptions involving biological, psychological or social causes and “effects” that may improve or worsen health, which are simplistic in accounting for the multifactorial etiology of health [28]. We find that each issue influences perceived health status in isolation, which is only weakly predictive of perceived health status. This is the case, for example, for major life events, which explain only about 9% of variance in the subsequent development of a cancerous disease [29].

Comorbidity of psychiatric and somatic disorders is generally associated with many social harms, such as stigmatization and, due to mental disorders, with low autonomy. Conversely, self-determination, or the power to decide for oneself (empowerment), may be a protective factor against the development of various psychiatric and somatic disorders [30]. The items of the SCOOHPI scale that evaluate the physical or oral well-being dimensions are part of a search for information on the empowerment capacity and desire to control the situation with a physical problem, e.g., “I have a balanced diet”, “I think about washing myself (shower, bath, cleaning)”, “I eat healthy food” or “alcohol, tobacco, drugs have negative effects on my oral health”, or a potential oral problem, e.g., “I brush my teeth and/or my dentures”, “I neglect my oral health”, etc. Coping strategies affect healthy or risky behavior. Generally, emotion-based (or trying to avoid it like stress situation for example) coping is accompanied by risk-taking behavior, such as failure to recognize symptoms, poor adherence to therapy, lack of preventive behavior, and substance abuse. Conversely, problem-focused coping is expressed through active participation in care or compliance with care. However, if the illness is too long-lasting or uncontrollable, such as schizophrenia, the beneficial effects of problem-focused coping strategies may be ineffective [31].

With regard to moral well-being strategies, studies have shown that perceived stress and its control have an impact on the improvement of coping strategies and on the adoption of healthy or risky behavior [32,33]. The SCOOHPI provides information about the control of perceived stress, e.g., “I am looking for simple pleasures (walk, drink coffee, listen to music, watch TV, etc.)”, or, conversely, “I’m afraid to go to the dentist”, – as well as coping strategies used for health (“I manage to visit my dentist”), and risky behavior, e.g., “I forget to brush my teeth”.

Determining the external validity of the scale is an essential step by which we validate whether the studied scale is correlated with other scales assessing the same concept. No SCOOHPI item is well correlated (r > 0.55) with any Brief-COPE item. The Brief-COPE scale is a generic coping scale [24]. However, the SCOOHP scale, which explores coping strategies, is both specific to oral health and to schizophrenia. These scales address different concepts, and, therefore, we cannot establish correlations. This demonstrates the complexity of constructing tools to assess global health, which refer to measures that give a broad view of health, not referring to specific problems, such as cancer, diabetes, dental problems, or mental disorders; this tool focused on global measures of perceived physical and mental health [34]. Further studies are needed to clarify these points.

## 5. Limitations

One of the difficulties in measuring perceived health lies in the dissociation between the reality of the disease, the intensity of the response, and the adequacy of the perception. This difficulty is all the more marked in people suffering from schizophrenia, in which the presence of psychotic elements or cognitive disorders calls into question the ability of these people to document their own health status [5]. Moreover, the concept of global health, as defined over the last 10 years, includes physical, mental and oral health, and takes into account the environment in which people live, confounding the complexity of the construction of tools that measure perceived health.

The social isolation measures imposed by the COVID-19 pandemic, with disruptions in the care of PWS, generated stress and anxiety. This factor impacted questionnaire responses through this period.

Although we established the internal structure validation of the SCOOHPI scale, as well as the reproducibility of its results, the sensitivity to change (test–retest) and reliability in other PWS populations must continue to be studied.

## 6. Conclusions

The difficulty and discrimination indices of 18 items of the SCOOHPI scale are acceptable according to the Rasch model, as are the inter-item (0.25) and inter-score (α = 0.85) correlations. This scale is considered an index, yielding an overall score between 0 and 72, with a five-modality Likert scale of response. It is also a profile with the following three dimensions of oral health-related coping clearly emerging from each other: physical well-being strategies (α = 0.72), moral well-being strategies (α = 0.60), and oral well-being access strategies (α = 0.79). The validation study of the SCOOHPI scale is still ongoing, investigating the reproducibility of the results of this scale, its sensitivity to change, and its reliability in other PWS. The SCOOHPI scale reveals new perspectives in terms of understanding oral health behavior/global health, as well as individualized cognitive and behavioral treatments to accompany PWS for oral health care.

## Figures and Tables

**Table 1:** Item correlation with a Global Score of SCOOHP 23 Items.

SCOOHP items	1	2	3	4	5	6	7	8
Item-scale correlations	0.601	0.502	0.488	0.528	0.487	−0.036	0.130	0.506
SCOOHP items	9	10	11	12	13	14	15	16
Item-scale correlations	0.061	0.463	0.714	0.529	−0.171	0.008	0.495	0.476
SCOOHP items	17	18	19	20	21	22	23	
Item-scale correlations	0.538	0.660	0.585	−0.173	0.112	0.234	−0.125	

SCOOHP: Schizophrenia Coping Oral Health Profile.

**Table 2:** Rasch analysis for SCOOHP (23 items).

Item		Missing	Mean	SD	Skew	Kurtosis	W(p)	Item Difficulty	Item Discrimination	α If Deleted
1	I am looking for simple pleasures (walk, drink coffee, listen to music, watch TV …).	5.88%	3.6	1.22	−0.8	−0.23	0.85 (0.000)	0.72	0.43	0.85
2	I go out of my home	11.76%	3.13	1.36	−0.27	−0.16	0.89 (0.000)	0.63	0.37	0.85
3	I eat for my pleasure	6.86%	2.59	1.18	0.73	−0.21	0.86 (0.000)	0.52	−0.28	0.82
4	I have a hobby (music, singing, drawing, movie, and ballads …)	5.88%	3.08	1.33	−0.24	−1.02	0.89 (0.000)	0.62	0.28	0.85
5	When I move, I feel good	6.86%	3.49	1.21	−0.48	−0.81	0.88 (0.000)	0.70	0.49	0.85
6	I feel trapped by my relationship with sugar	6.86%	3.52	1.54	−0.53	−1.22	0.81 (0.000)	0.70	0.27	0.86
7	I have my own medicines to manage my health	6.86%	3.28	1.42	−0.25	−1.19	0.88 (0.000)	0.66	0.01	0.81
8	I have a balanced diet	7.84%	3.43	1.21	−0.47	−0.69	0.89 (0.000)	0.69	0.42	0.85
9	I snack between meals	6.86%	3.05	1.27	0.09	−0.92	0.90 (0.000)	0.61	0.15	0.81
10	When I am stressed or don’t feel good, I eat Iess, or I eat more	6.86%	2.84	1.33	0.19	−1.10	0.90 (0.000)	0.57	−0.02	0.81
11	I think about washing myself (shower, bath, cleaning)	5.88%	3.93	1.35	−1.16	0.11	0.76 (0.000)	0.79	0.74	0.83
12	I brush my teeth and/or my denture	6.86%	3.36	1.48	−0.36	−1.23	0.85 (0.000)	0.79	0.75	0.83
13	I neglect my oral health	5.88%	3.24	1.48	−0.16	−1.38	0.86 (0.000)	0.67	0.48	0.85
14	I brush my tongue	6.86%	2.58	1.55	0.42	−1.29	0.82 (0.000)	0.52	0.19	0.81
15	I take care of my mouth to have a good breath	6.86%	3.17	1.38	−0.26	−1.15	0.89 (0.000)	0.65	0.65	0.84
16	I take care of my mouth to have a good dentition	5.88%	3.27	1.43	−0.18	−1.34	0.87 (0.000)	0.63	0.63	0.84
17	I eat healthy food	6.86%	3.63	1.19	−0.63	−0.49	0.87 (0.000)	0.65	0.49	0.85
18	I think about drinking water (normal or sparkling) when my mouth is dry	5.88%	3.51	1.42	−0.54	−1.12	0.84 (0.000)	0.73	0.60	0.84
19	I can coordinate the movement of my hands in order to brush my teeth	6.86%	3.76	1.6	−0.92	−0.85	0.71 (0.000)	0.75	0.63	0.84
20	I forget to brush my teeth	7.84%	3.21	1.34	−0.1	−1.11	0.89 (0.000)	0.64	0.54	0.84
21	Alcohol, tobacco, drugs have negative effects on the oral health	9.80%	3.36	1.65	−0.4	−1.51	0.80 (0.000)	0.67	0.10	0.86
22	I manage to visit my dentist	6.86%	2.73	1.55	0.24	−1.45	0.84 (0.000)	0.55	0.20	0.86
23	I’m afraid to go to the dentist	5.88%	3.74	1.43	−0.74	−0.86	0.80 (0.000)	0.75	0.30	0.85

SD: standard deviation; mean inter-item-correlation = 0.15; Cronbach’s α=0.80. Items with negative discrimination (items 3 and 10) or close to 0 (items 7, 9 and 14) were removed from the questionnaire. The SCOOHP was reduced from 23 to 18.

**Table 3:** Item correlation with the global score of the SCOOHPI 18 items.

SCOOHPI items	1	2	4	5	6	8	11	12	13
Item-scale correlations	0.62	0.53	0.49	0.50	−0.21	0.53	0.79	0.57	−0.21
SCOOHPI items	15	16	17	18	19	20	21	22	23
Item-scale correlations	0.51	0.50	0.62	0.67	0.66	−0.22	−0.11	0.20	−0.16

SCOOHPI: Schizophrenia Coping Oral Health Profile and Index.

**Table 4:** Rasch analysis of the dimension 1 of the SCOOHPI.

Item	Missing	Mean	SD	Skew	Kurtosis	W(p)	Item Difficulty	Item Discrimination	α if Deleted
5	6.86%	3.49	1.21	−0.48	−0.81	0.88 (0.000)	0.70	0.46	0.68
8	7.84%	3.43	1.21	−0.47	−0.69	0.89 (0.000)	0.69	0.47	0.68
11	5.88%	3.93	1.35	−1.16	0.11	0.76 (0.000)	0.79	0.67	0.61
17	6.86%	3.63	1.19	−0.63	−0.49	0.87 (0.000)	0.73	0.54	0.66
19	6.86%	3.76	1.6	−0.92	−0.85	0.71 (0.000)	0.75	0.59	0.63
21	9.80%	3.36	1.65	−0.4	−1.51	0.80 (0.000)	0.67	0.12	0.79

SD: Standard deviation; Mean inter-item-correlation = 0.319; Cronbach’s α = 0.7185.

**Table 5:** Rasch analysis of the dimension 2 of the SCOOHPI.

Item	Missing	Mean	SD	Skew	Kurtosis	W(p)	Item Difficulty	Item Discrimination	α if Deleted
1	5.88%	3.6	1.22	−0.8	−0.23	0.85 (0.000)	0.72	0.35	0.56
2	11.76%	3.13	1.36	−0.27	−0.16	0.89 (0.000)	0.63	0.58	0.45
4	5.88%	3.08	1.33	−0.24	−1.02	0.89 (0.000)	0.62	0.42	0.53
20	7.84%	3.21	1.34	−0.1	−1.11	0.89 (0.000)	0.64	0.24	0.60
22	6.86%	2.73	1.55	0.24	−1.45	0.84 (0.000)	0.55	0.30	0.58
23	5.88%	3.74	1.43	−0.74	−0.86	0.80 (0.000)	0.75	0.18	0.62

SD: Standard deviation; Mean inter-item-correlation = 0.207; Cronbach’s α = 0.604.

**Table 6:** Rasch analysis of the dimension 3 of the SCOOHPI.

Item	Missing	Mean	SD	Skew	Kurtosis	W(p)	Item Difficulty	Item Discrimination	α if Deleted
6	6.86%	3.52	1.54	−0.53	−1.22	0.81 (0.000)	0.70	0.28	0.82
12	6.86%	3.36	1.48	−0.36	−1.23	0.85 (0.000)	0.67	0.74	0.71
13	5.88%	3.24	1.48	−0.16	−1.38	0.86 (0.000)	0.65	0.51	0.76
15	6.86%	3.17	1.38	−0.26	−1.15	0.89 (0.000)	0.63	0.67	0.73
16	5.88%	3.27	1.43	−0.18	−1.34	0.87 (0.000)	0.65	0.68	0.72
18	5.88%	3.51	1.42	−0.54	−1.12	0.84 (0.000)	0.70	0.42	0.78

SD: Standard deviation; Mean inter-item-correlation = 0.389; Cronbach’s α = 0.789.

**Table 7:** Correlations between each dimension (SCOOHPI-Brief COPE).

	SCOOHPI Dim * 1	SCOOHPI Dim * 2	SCOOHPI Dim * 3
Brief COPE item 1	0.38	0.10	0.09
Brief COPE item 2	0.08	0.15	0.10
Brief COPE item 3	0.17	0.15	0.04
Brief COPE item 4	0.21	0.10	−0.01
Brief COPE item 5	0.09	0.00	0.07
Brief COPE item 6	0.26	0.10	0.06
Brief COPE item 7	0.13	0.20	0.09
Brief COPE item 8	0.09	−0.06	0.00
Brief COPE item 9	0.07	−0.19	−0.16
Brief COPE item 10	−0.03	−0.12	0.01
Brief COPE item 11	0.14	−0.02	0.02
Brief COPE item 12	0.07	0.04	0.09
Brief COPE item 13	0.11	0.00	−0.05

Dim *: dimension. No SCOOHPI item correlated well (r > 0.55) with any Brief-COPE item either between overall score means or dimensional score means for each variable.

## Data Availability

The data that support the findings of this study are available from the corresponding author upon reasonable request.
